# The SARS-CoV-2 Pandemic Does Not Absolve From Solid Medical Trade

**DOI:** 10.31486/toj.21.0089

**Published:** 2021

**Authors:** Josef Finsterer, Claudia Stöllberger

**Affiliations:** ^1^ Klinik Landstrasse, Messerli Institute, Vienna, Austria; ^2^ Klinik Landstrasse, Medical Department, Vienna, Austria

## TO THE EDITOR

With interest, we read the article “Suspected COVID-19-Induced Myopericarditis” by Okor et al about a 72-year-old female with a history of arterial hypertension and chronic obstructive pulmonary disease who was admitted 1 week after testing positive for severe acute respiratory syndrome coronavirus 2 (SARS-CoV-2) because of worsening shortness of breath.^1^ During hospitalization, the patient developed chest pain, systolic dysfunction, and cardiogenic shock attributed to suspected myopericarditis.^1^ The study is appealing but prompts comments and concerns.

The main limitation of the study is that the suspected diagnosis of myopericarditis was neither confirmed by imaging (cardiac magnetic resonance imaging [MRI] with contrast medium [late gadolinium enhancement]) nor by pathologic investigations (endomyocardial biopsy or autopsy).^1^ Diagnosing myopericarditis solely upon clinical presentation, laboratory tests, transthoracic echocardiography, and the putative effect of treatment is insufficient.

Echocardiographic images in Figure 2 strongly suggest the presence of Takotsubo syndrome (TTS).^1^ TTS could explain chest pain, elevation of troponin and pro-brain natriuretic peptide, the electrocardiogram changes, the echocardiographic findings, and even the cardiogenic shock. Thus, echocardiographic investigations should be revised for the presence/absence of TTS.

A further limitation of the study is that myocardial infarction was not excluded by coronary catheter angiography or coronary computed tomography. When suspecting TTS, it is crucial to demonstrate that systolic dysfunction is not due to coronary heart disease.

A further limitation is that nothing is reported about the degree of lung involvement and about right ventricular function. We should be told if the patient required artificial ventilation because of cardiac compromise or because of SARS-CoV-2 pneumonia or acute respiratory distress syndrome. Elevated inflammatory parameters are more likely attributable to pulmonary infection than to myocarditis.

On hospital day 4, the patient is reported to have become agitated, disorientated, and confused. Because cerebral involvement is a frequent complication of SARS-CoV-2 infections,^2^ we should know if the patient underwent neurologic workup or not. Of particular interest are the results of cerebral MRI, electroencephalography, and eventually cerebrospinal fluid investigations. Is it conceivable that systolic dysfunction was attributable to brain-heart interactions? Emotional stress is a well-appreciated trigger of TTS,^3^ and SARS-CoV-2 is frequently associated with TTS.^4^

The rationale for application of colchicine is unclear. Colchicine for putative SARS-CoV-2–associated myopericarditis is ineffective, particularly if no proactive anti-inflammatory therapy is given prior to colchicine.^5^ The patient was treated on hospital day 1 with azithromycin, ceftriaxone, and prednisolone. We should be told why 2 antibiotics were applied simultaneously.

Overall, the case report has several limitations which challenge the results and their interpretation. The diagnosis of myopericarditis remains unsupported. The patient most likely had SARS-CoV-2–associated TTS, and inflammatory parameters were attributable to the pulmonary infection. The pandemic is no reason to refrain from thorough diagnostic workup and appropriate exclusion of differentials.
